# Advances of Nanomedicine in Radiotherapy

**DOI:** 10.3390/pharmaceutics13111757

**Published:** 2021-10-21

**Authors:** Wei Liu, Bo Chen, Haocheng Zheng, Yun Xing, Guiyuan Chen, Peijie Zhou, Liting Qian, Yuanzeng Min

**Affiliations:** 1 Department of Radiation Oncology, The First Affiliated Hospital of USTC, Division of Life Sciences and Medicine, University of Science and Technology of China, Hefei 230001, China; liuweizkdfyy@ustc.edu.cn (W.L.); zhpejie@ustc.edu.cn (P.Z.); 2 Department of Bio-X Interdisciplinary Science at Hefei National Laboratory (HFNL) for Physical Science at the Microscale, University of Science and Technology of China, Hefei 230026, China; cb0216@mail.ustc.edu.cn (B.C.); minyz@ustc.edu.cn (Y.M.); 3 Department of Endocrinology, The First Affiliated Hospital of USTC, Anhui Provincial Hospital, Division of Life Sciences and Medicine, University of Science and Technology of China, Hefei 230026, China; zhenghc233@mail.ustc.edu.cn (H.Z.); xy0408@mail.ustc.edu.cn (Y.X.); cgy731@mail.ustc.edu.cn (G.C.); 4 CAS Key Lab of Soft Matter Chemistry, University of Science and Technology of China, Hefei 230026, China; 5 School of Chemistry and Materials Science, University of Science and Technology of China, Hefei 230026, China

**Keywords:** radiotherapy, radiosensitization, nanoparticles, drug delivery, immunotherapy

## Abstract

Radiotherapy (RT) remains one of the current main treatment strategies for many types of cancer. However, how to improve RT efficiency while reducing its side effects is still a large challenge to be overcome. Advancements in nanomedicine have provided many effective approaches for radiosensitization. Metal nanoparticles (NPs) such as platinum-based or hafnium-based NPs are proved to be ideal radiosensitizers because of their unique physicochemical properties and high X-ray absorption efficiency. With nanoparticles, such as liposomes, bovine serum albumin, and polymers, the radiosensitizing drugs can be promoted to reach the tumor sites, thereby enhancing anti-tumor responses. Nowadays, the combination of some NPs and RT have been applied to clinical treatment for many types of cancer, including breast cancer. Here, as well as reviewing recent studies on radiotherapy combined with inorganic, organic, and biomimetic nanomaterials for oncology, we analyzed the underlying mechanisms of NPs radiosensitization, which may contribute to exploring new directions for the clinical translation of nanoparticle-based radiosensitizers.

## 1. Introduction

Although great efforts have been made in biology theories and medical technologies in recent years, cancer remains the leading threat to human lives. According to the latest report, it was estimated that there were 19.3 million new cancer cases and almost 10.0 million cancer deaths in 2020 [1]. Since radiotherapy (RT) was first used in the medical area in 1896, it has been developed for the following 100 years and eventually became one of the mainstream clinical strategies together with chemotherapy and surgery.

**Figure 1:**
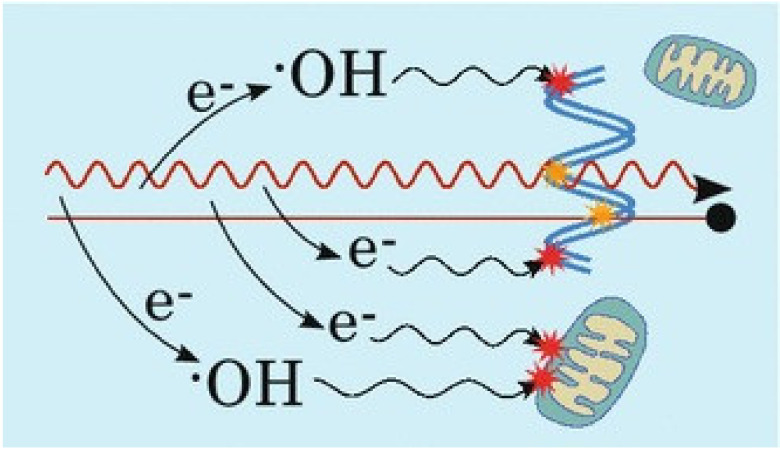
Illustration of mechanisms of radiation damage. Both photon and ion radiation (red wiggly and straight lines, respectively) may directly damage DNA (marked with yellow stars) or other parts of the cell, such as mitochondria (damage not shown), as well as ionize the medium, thereby producing radicals and other reactive species (represented here by the •OH radical) as well as secondary electrons, which can cause indirect damage after diffusion (red stars). Secondary electrons may also react with the medium to further increase the number of radicals. Reproduced with the permission from Sung et al., CA Cancer J Clin; published by Springer, 2016.

A series of complicated reactions will occur inside the tumor after irradiation (Figure 1) [2], and they can be mainly concluded as three stages chronologically. During the initial physics stage, the high-energy photons or ions would interact with the biological medium, depositing energy. They can either directly kill the cancer cells by fracturing the DNA through ionization or generate secondary species such as low-energy electrons or radicals to further damage DNA. Current researchers mostly evaluate the efficiency of RT by monitoring the amount of DNA strand breakages produced. Several relevant researchers show that 70% of the DNA damage is caused by radicals or other active species such as •OH, NO•, H•, and H_2_O_2_. These highly reactive species, particularly •OH, will rupture molecule bonds and DNA or proteins of cellular structures, which would undoubtedly influence the stability of cell membranes and organelles such as mitochondria. The rest of the RT-related DNA damage is a result of DNA’s direct fragmentation and secondly electrons’ interaction. Through a process called “dissociative electron attachment”, the secondly electrons can lead to the DNA strand breaks. They can also further interact with the water medium to produce more radicals. These indirect damages caused by secondary species occur at the chemical stage. In the end, the damaged cells expose the consequences of being exposed to irradiation during the biologic stage. For example, a high enough dose of radiation may cause cancer cells’ mitotic senescence, catastrophe, and even apoptosis or necrosis.

Radiation therapy can be mainly divided into two types: external beam radiotherapy (EBRT) and internal radioisotope therapy (RIT). As far as EBRT is concerned, external high-energy radiation such as proton or electron beams can induce cancer cells’ death without being limited by the penetrating depth [3]. Thus, it has been widely used in clinically treating various local solid tumors (such as breast, lung, esophageal, colorectal, liver and prostate tumors). However, for internal RIT, therapeutic radioisotopes would usually be introduced into the tumor through minimally invasive methods. For example, directly intratumor injecting radioisotopes and intravenous injecting liposomes, antibodies, or nanoparticles containing tumor-targeting ligands as suitable tumor-homing carriers are both feasible [3,4,5]. Thus, RIT can treat not only local solid tumors but also metastatic tumors [6]. In the current clinical treatment of cancer, nearly half of patients will receive RT alone or RT-based combination therapy.

However, current clinical RT often fails to achieve the expected effect because of the restriction of many factors. First of all, during EBRT, tumor as a soft tissue has a relatively low radiation absorption rate. This leads to the fact that a high dose of radiation is often needed to produce sufficient killing effect in the actual RT process, which will undoubtedly cause damage to adjacent normal tissues. As for RIT, the targeted delivery of radioactive isotopes into tumor sites in vivo can be achieved in the presence of corresponding tumor homing carriers. In this way, the possible caused systemic radioactive toxicity to normal organs cannot be ignored. Additionally, some inherent characteristics of solid tumors also greatly limit the RT’s efficacy. Different from normal tissues, the tumor microenvironment usually has the following features: the asymmetrical distribution of nutrients, slightly acidic pH, insufficient oxygenation, and higher levels of reactive oxygen species [7]. These features contribute to tumor invasion, metastasis, and recurrence, as well as resistance to various therapeutic strategies, including RT.

As a multi-disciplinary field, nanotechnology has become one of the most promising research directions in the treatment of malignant tumors in recent decades. Nanomaterials range in size from a few nm to 100 nm in two or three dimensions. Furthermore, because of their unique physical and chemical properties and easy chemical modification, nanomaterials have been used in more and more related studies to enhance the radiation response and overcome the radiotherapy tolerance of tumors, and then finally achieve enhanced RT (Table 1) [8]. For example, due to the Compton scattering effect, nanomaterials containing high atomic number elements such as Au, Pt, Ba, and Bi have higher mass energy absorption coefficients for X-rays; thus, they can serve as a sensitization agent to deposit radiant energy within tumors more effectively during RT [9]. In this way, the toxic and side effects to adjacent normal tissues can be reduced significantly while increasing radiation damage to tumors. In addition, nanomaterials can also act as carriers to deliver a large number of therapeutic molecules into tumors through active or passive targeting. The therapeutic molecules include, but are not limited to, chemotherapeutics, photosensitizers, gases, RNA, photothermal agents, therapeutic radioisotopes, immunotherapy adjuvants, and immune checkpoint inhibitors. Therefore, the combined anti-tumor strategy of radiotherapy and chemotherapy, hyperthermia, immunotherapy, or other therapies can also be realized. More interestingly, nanotechnology provides a variety of novel methods to adjust the tumor microenvironment (TME) in solid tumors, which will be helpful to overcome hypoxia-related radiation tolerance, thus enhancing RT efficiency.

## 2. Nanomaterials as Radiotherapy Sensitizers

**Figure 2:**
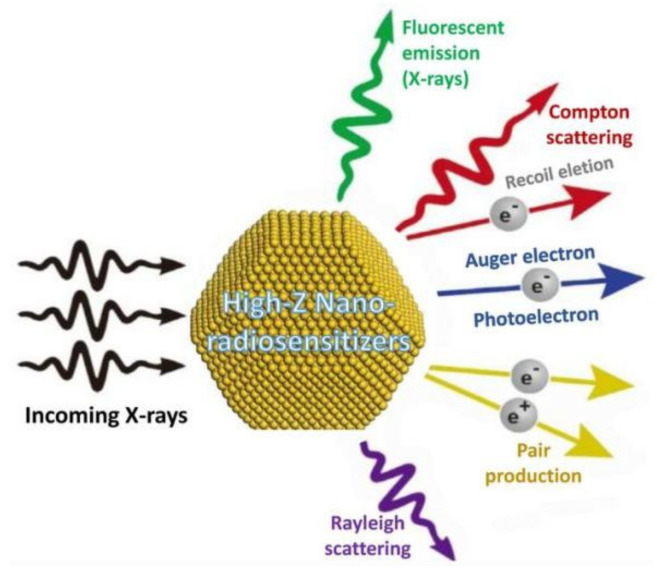
Interaction between X-rays and high-Z element material nanoparticles. Reproduced with the permission from Gong et al., Wuli Huaxue Xuebao/Acta Phys. -Chim. Sin; published by Wuli Huaxue Xuebao/Acta Phys. -Chim. Sin, 2018.

In the beginning, we need to introduce the relevant mechanisms of nanomaterials acting as RT sensitizers. When the material is being irradiated by X-rays, a series of physical processes would happen, such as Rayleigh scattering, photoelectric effect, Compton scattering, and others (Figure 2) [24]. During Rayleigh scattering, the photon in the X-ray collides with the relevant atom in an elastic way, which almost does not deposit any radiation energy in the tissue and thus has no enhancement effect on RT. As for the photoelectric effect, the inner electrons excited by incident X-rays would be emitted and move a certain distance (hundreds of microns), thus causing certain damage to the nearby tissue. Since the intensity of photoelectric effect is proportional to (Z/E) [3] (E means energy of X-rays while Z represents the material’s atomic number), the photoelectric effect of noble metal materials such as Pt (Z = 78) is much stronger than that of biological tissues (average Z = 7.4). Electrons from higher orbitals will occupy the vacancies caused by the ejected photoelectrons in the material’s atoms. The excess energy generated by such an electron rearrangement in atomic orbitals would be released by emitting fluorescent photons or Auger electrons, which can travel short distances (usually about 10 nm) and produce strong ionization and large numbers of free radical molecules in local areas. However, the interacting atom must be close enough to the target molecule in order to make good use of the above Auger effect. In Compton scattering (inelastic interactions), a part energy of the incident X-rays would transfer to the collided atoms’ electrons, which are then ejected at a certain angle. The free radicals and secondary electrons generated in this physical process are of great significance for enhancing the tumor cell-related DNA damage during RT.

Reactive oxygen species (ROS) are very important in enhancing RT efficiency. As is known to all, metabolic wastes such as free radicals or ROS are constantly generated during cell metabolism. ROS can induce the mutations and the breaks of single or double strands in DNA molecules, which are often harmful to normal cells. To maintain normal cellular metabolic processes, a complex antioxidant mechanism has been developed to remove excess ROS in cells. However, for cancer cells, excessive metabolism can lead to a significant increase in intracellular ROS level. Otherwise, the high-energy radiation can also react with water or oxygen molecules to produce more ROS during RT. Therefore, maybe we can consider adopting appropriate materials to further increase the ROS level and inhibit the antioxidant mechanism in tumor cells, thus leading to more serious RT-related DNA damage and finally enhancing RT’s effect.

An important premise of the above strategies is the effective enrichment of materials at tumor sites, which is mainly achieved via passive or active targeting ability of nanomaterials. The abnormal formation of blood vessel walls and underdeveloped lymphatic system around tumor tissues limits macromolecules’ influx from tumors, leading to the accumulation of nanoparticles in tumor tissues through an enhanced permeability and retention effect. Furthermore, the nanomaterials’ PEGylation can also effectively increase their blood circulation time and tumor intake (hormones, glucose molecules, etc.) to the surface of the nanomaterial, so as to avoid the excessive dependence on passive targeting. Meanwhile, active targeting is achieved by attaching other molecules (such as antibodies, folic acid, hormones, glucose molecules, etc.) to the surface of the nanomaterial, so as to avoid the excessive dependence on passive targeting.

As a result of these unique physicochemical properties of nanomaterials and their excellent radiotherapy sensitization potential shown in many clinical experiments over the past decades, many kinds of nanoparticles or other nanostructures have been widely used as RT sensitizers.

### 2.1. Gold Nanoparticles

Due to its high chemical inertness, easy modification, good biosecurity, high delivery efficiency, and other physical or chemical properties, gold (Z = 79) nanoparticles were firstly and most widely used in the radiosensitization field [25,26]. They have been used to synthesize nanostructures that have different sizes and shapes. For example, spherical nanoparticles, hollow nanoshells, nanocubes, and nanorods are all able to be constructed in a precisely controlled way. The mechanism of radiosensitization of gold nanoparticles has also been extensively studied. In the earlier study, Sanche et al. demonstrated that gold nanoparticles’ radiosensitization effect is achieved mainly through enhancing the associated DNA damage [27]. For instance, it was found that gold nanoparticles’ presence can induce an accelerated fragmentation of DNA caused by X-ray irradiation. Further studies also showed that the RT sensitization effect of gold nanoparticles is related to their size, surface modification, and location distribution [28,29], and the radiotherapy sensitization of gold nanoparticles shows a significant size dependence within certain limits, because the size may lead to an uneven distribution of gold atoms in the nanoparticles and then affect the secondary ionization during the interaction between the gold nanoparticles and X-ray. Related studies have also proved that the surface modifications of gold NPs and their distribution within tumor cells also influence the radiosensitization ability, but the exact physical mechanism remains to be studied. Otherwise, Hainfeld et al. also found that they have the ability to act as contrast agents for X-ray and MRI for tumor detection [30,31].

**Figure 3:**
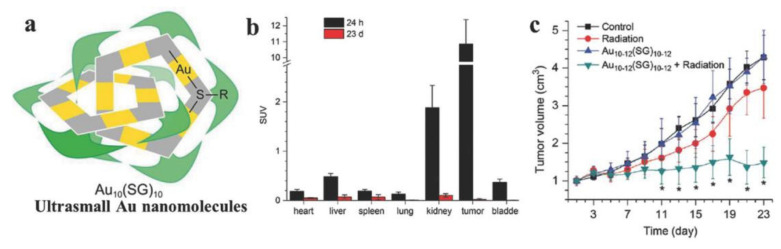
Au nanoclusters as radiosensitizer. (**a**) Structure schematic illustration of ultra-small Au nanoclusters. (**b**) Biodistribution of Au_10–12_ (SG) _10–12_ at 24 h and 23 days p.i. (**c**) Tumor growth curves of mice with different treatments. It showed that Au nanoclusters could efficiently increase the radiotherapeutic responses of tumor under irradiation. Reproduced with the permission from Zhang et al., Adv. Mater.; published by John Wiley and Sons, 2014. The star denotes significant difference from the control group (*p* < 0.05).

Despite the good biocompatibility and high chemical inertness, the possible toxic and side effects of gold nanoparticles as RT sensitizers in vivo still need to be seriously considered. Nanoparticles smaller than 5 nm in size can be eliminated rapidly in vivo through renal clearance, while their accumulation in tumors is also low. After intravenous administration, the accumulation of larger nanoparticles ranging from 20 to 100 nm in spleen or liver may last over a few months, which may lead to potential long-term toxicity. Therefore, Zhang et al. developed polymer micelles loaded with ultra-small gold nanoparticles (1.9 nm) in order to keep a good balance between the kidney’s ability to clear gold nanoparticles and their ability to target tumors (Figure 3) [32]. Compared with the ultra-small nanoparticles alone, the polymer micelles had a much higher tumor intake and longer circulation time. Moreover, they can not only greatly improve the RT efficiency but also be dissociated into ultra-small gold nanoparticles and then be effectively removed from the body, avoiding possible long-term toxicity.

### 2.2. Rare Earth Nanoparticles

**Figure 4:**
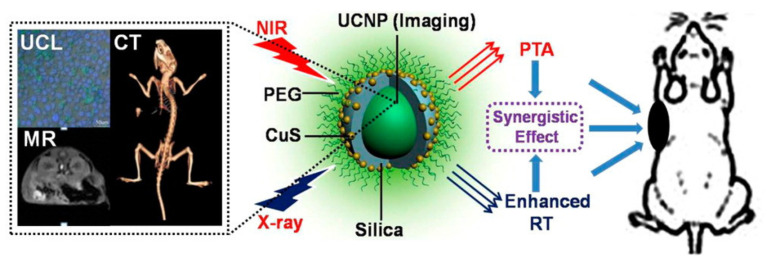
Schematic of a core/satellite nanotheranostic (CSNT) as a radiosensitizer. This CSNT is constructed by decorating ultra-small CuS nanoparticles onto the surface of a silica-coated rare earth upconversion nanoparticle (UCNP). UCNP cores are used to enlarge the local radiation dose for the enhanced RT, and CuS satellites are responsible for converting the 980 nm laser into heat for photothermal ablation (PTA). The combination of PTA and CSNT-enhanced radiotherapy (RT) could give rise to a strong synergistic effect and then construct an RT/PTA synergistic system. Reproduced with the permission from Xiao et al., J. Am. Chem. Soc.; published by American Chemical Society, 2013.

Rare earth elements whose Z numbers range from 57 to 71 have also been widely used in enhancing RT. For example, Gadolinium-based (Gd, Z = 67) nanoparticles are being explored as the potential RT sensitizers due to their large atomic number, low toxicity, and rapid removal from the body. In addition, the chelates of gadolinium can act as clinical MR contrast agents. For instance, ultra-small gadolinium oxide@polysiloxane core–shell NPs synthesized by Tillement could effectively become enriched within tumors after intrapulmonary nebulization or intravenous injection, greatly improving the survival of mice through the strong radiosensitizing effects [33]. In the meantime, the safety of those nanoparticles is guaranteed by renal clearance. Moreover, Gd atoms can emit distant X-rays, γ-rays, Auger electrons, and internal converted electrons when being irradiated by neutrons. So, nanomaterials containing gadolinium can be used for enhanced neutron capture therapy [34]. As a result of its much higher neutron capture cross-section area compared with boron atoms, Gd-based neutron capture therapy has been proven to be significantly better than neutron capture therapy based on boron, which is currently used clinically. Nanoparticles based on Thulium (Z = 69), Ytterbium (Z = 70), or other rare earth elements have also been developed for enhanced RT. For example, the Shi group creatively synthesized a core/satellite nanostructure made up with UNCPs and ultra-small CuS nanoparticles-decorated silica shells. It could induce highly localized radiation energy to enhance RT and convert the NIR into heat for photothermal therapy (PDT). Moreover, these nanomaterials may have the potential for trimodal imaging based on CT/MR/upconversion luminescence (Figure 4) [35].

### 2.3. Photon Beam Radiotherapy and Ion Beam Radiotherapy

**Figure 5:**
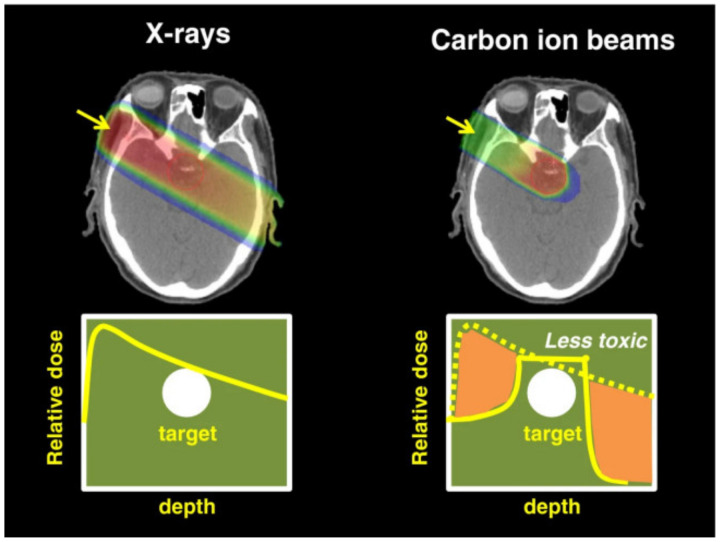
The advantage of carbon ion beams compared with X-rays through a better dose distribution. As a result of the “Bragg Peak” effect mentioned above, carbon ion beams allow a highly localized deposition of energy, which can be utilized for increasing radiation doses to tumors while minimizing irradiation to adjacent normal tissues (personalized cancer treatment). Reproduced with the permission from Ohno, EPMA J.; published by Spring, 2013.

Furthermore, EBRT can be divided into photon beam radiotherapy and ion beam radiotherapy according to the type of radiation source used. The former usually uses photon beams such as X-rays or γ-rays, which can effectively ionize lipids, DNA, and other biological molecules to produce plenty of free radicals. Photon-based RT has already been utilized to treat cancer clinically for decades because of the relatively simple devices, and it has achieved great successes. However, due to the exponential relationship between the radiation dose deposition of the high-energy photon beam and the tissue depth, a considerable part of the total radiation dose will be absorbed by the normal tissue near the tumor and cause damage. In addition, the shape features of the tumor make it difficult to irradiate the whole, which may cause tumor recurrence. In order to overcome the above shortcomings, radiation therapy based on ion beams is proposed as an alternative, using an ion beam as the radiation source such as hydrogen ion (proton therapy), helium ion, carbon ion, and oxygen ion (heavy ion therapy). As charged particles, the tissue-penetrating ability of protons is just influenced by its energy [36]. Compared with photons, protons have the physical advantages of depositing their major energy at the “Bragg peak”. Therefore, distal or proximal normal tissues can both be protected as a result of greatly reduced radiation doses. For example, in comparation with the most advanced volumetric modulated arc RT or intensity-modulated RT, proton therapy can deliver higher doses of radiation into tumor volumes while reducing radiation dose deposited in “total body” by about 50–60%. As for heavy ion therapy, it has a similar physics rationale with protons and its own advantage. Take carbon ion radiation therapy (CIRT) as an example. Firstly, because of the greater mass than protons, carbon ions’ multiple Coulomb scattering is inhibited during its movement (Figure 5) [37], causing higher relative bioavailability and more serious DNA damage than proton therapy. Secondly, carbon ions can lead to more direct double-stranded DNA damage due to its higher linear energy transfer than protons [38]. Thus, heavy ion radiation therapy has been proven to be more effective when treating RT-resistant cancers such as head and neck cancer, prostate cancer, and so on.

### 2.4. Nanoparticles for Radiosensitization with Other Mechanisms

Relevant studies have shown that the X-ray absorption coefficient of a material meets the following formula:

$$\mu =\rho \;Z^{4}/\left(AE^{3}\right).$$

where *Z* represents the atomic number, *A* means the atomic mass, and *E* refers to the energy of the X-rays. It is obvious that the absorption coefficient has a close relationship with the atomic number. So, materials containing a large *Z* number of elements can deposit the radiation energy into tumors more effectively. This is also the main reason why nanoparticles containing elements such as Au and rare earth elements can be used as sensitizers for RT. Otherwise, other types of nanomaterials can sensitize RT through some different mechanisms. For example, Fe_3_O_4_ and Fe_2_O_3_ nanoparticles can improve the cancer cells’ sensitivity to radiation via oxidative stress mechanisms [20]. More specifically, firstly, the EPR effect will cause iron oxide nanoparticles’ accumulation at tumor sites. Then, released Fe_3_^+^ or the active surface of the nanoparticles will catalyze the decomposition of endogenous H_2_O_2_ to release O_2_ and further generate highly reactive ROS through the Fenton reaction and Haber–Weiss reaction, finally realizing sensitized RT.

**Figure 6:**
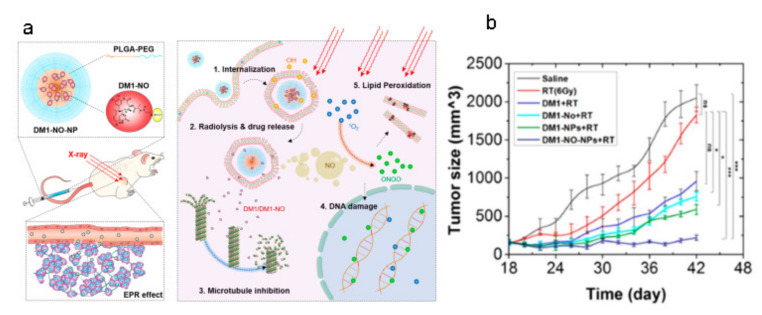
DM1-NO-encapsulated PLGA-b-PEG nanoparticles (DM1-NO-NPs) for enhanced radiation therapy. (**a**) The mechanism of DM1-NO-NPs as radiosensitizers. (**b**) Tumor growth curves of H1299 tumor-bearing nude mice, which received respectively i.v. injection of PBS, DM1, DM1-NO, DM1-NPs, or DM1-NO-NPs and then X-ray irradiation (6 Gy) after 4 h (*n* = 5). Significant tumor suppression was observed with animals in the DM1-NO-NPs+RT group. Reproduced with the permission from Gao et al., ACS Nano; published by American Chemical Society, 2020. * *p* < 0.05; *** *p* < 0.001; ns, no significant difference.

We can also enhance RT by selecting appropriate nanomaterials to effectively regulate the cell cycle so that tumor cells are in a more radiosensitive G2/M phase. Gao et al. first performed nitrification on maytansinoid (DM1) to obtain its prodrug DM1-NO, which was then loaded onto PLGA-b-PEG nanoparticles (Figure 6a) [39]. Before the above DM1-NO-encapsulated PLGA-b-PEG nanoparticles reach the tumor site through the EPR effect, the toxicity of DM1 was inhibited due to the nitrosylation and encapsulation of nanoparticles. However, when the nanoparticles were irradiated or within a low pH environment of endosome/lysosome, the S-N bond would break, leading to the release of DM1 and NO. On the one hand, DM1 can cause tumor cells to stagnate in the more radiation-sensitive G2/M phase by inhibiting microtubule assembly. On the other hand, the reaction between NO and ROS can generate free radicals such as peroxynitrite, which can cause DNA or lipid damage. The combination of these two effects can greatly improve RT efficiency (Figure 6b) [39].

In addition to the sensitized radiotherapy strategies mentioned above, enhancing RT through inhibiting the self-repair mechanism of DNA damage in cancer cells is also a possible way. It has been proven that the most important mechanism leading to tumor RT tolerance is the self-repair mechanism of tumor cells, which means that cancer cells can repair RT-related DNA damage. This repair mechanism is necessary for living cells to survive in an oxidized environment as metabolic wastes such as oxygen free radicals are constantly generated during normal physiological processes such as intracellular energy conversion, resulting in the corresponding oxidative DNA damage. For example, Mirjolet et al. demonstrated experimentally that TiO_2_ nanotubes can realize sensitized RT by inhibiting the DNA repair mechanism in tumor cells [40]. 

Different RT sensitization strategies can be combined to achieve the effect of “1 + 1 > 2”. For example, by encapsulating catalase (CAT) in a tantalum oxide (TaO_x_) nanoshell and further polyethylene glycolizing TaO_x_@Cat nanoparticles, Professor Liu’s group obtained a new RT sensitizer [41]. The enhancement of RT was achieved in a mouse tumor model without obvious side effects. The authors also summarized the possible reasons for TaO_x_@CAT-PEG’s excellent radiosensitization effect. First of all, tantalum can absorb X-rays strongly, so that radiation energy can be deposited into the tumor. Secondly, CAT loaded in a TaO_X_ hollow nanoshell can effectively improve the hypoxic microenvironment of the tumor by catalyzing the decomposition of endogenous H_2_O_2_ in a tumor microenvironment, further sensitizing RT. It also provides a new idea for us to carry on the related research in the future.

## 3. Nanomaterials for RIT

RIT has been introduced into oncology treatment through directly delivering therapeutic radionuclides into tumors [42,43,44]. Many recent studies have shown that the targeted delivery of radioisotopes via the utilization of nanoplatforms may effectively improve its bioavailability and minimize radiotoxicity to normal organs [45,46,47]. Moreover, RIT combined with other therapies and modulated by nanoparticle platforms has shown significant synergistic effect in recent studies [48,49].

### 3.1. Metal Nanoparticles

High-Z nanoparticles interacted with radiation released copious numbers of secondary particles, which can enhance radiation damage effects to tumor tissue [50]. Gaos synthesized the hybrid polymethacrylate grafted gold nanoparticles (PMAA-AuNPs) to synergize with ^131^I-mediated RT. The results of cell and animal experiments both indicated that PMAA-AuNPs could improve the tumor-killing effect of systemic ^131^I-mediated RT [51]. In another recent study, Tian et al. labeled ^131^I to human serum albumin-bound MnO_2_ NPs to establish a novel nanomedicine platform. Due to its strong permeability and good retention effect, ^131^I-HSA-MnO_2_ NPs are more likely to be uptaken by tumor tissues. The acidic microenvironment will cause ^131^I-HSA-MnO_2_ NPs’ degrading into ^131^I-HSA, thereby promoting nanoparticles’ penetration into tumor tissues. In addition, MnO_2_ can improve the hypoxia in TME, thereby enhancing the sensitivity of tumor cell to radionuclides ^131^I [52].

### 3.2. Non-Metallic Inorganic Nanoparticles

Yang constructed folic acid-coupled selenium nanoparticles (FA@SeNPs). The synergistic treatment of the FA@SeNPs and radioactive ^125^I can influence the cell cycle distribution, activate mitogen-activated protein kinase and p53 signal pathways, and promote tumor cell apoptosis [53]. In another instance, Tian et al. synthesized calcium bisphosphonate (CaBP-PEG) nanoparticles, which combined with radionuclide therapy can offer an excellent anti-tumor synergistic effect. Bisphosphonates can inhibit angiogenesis and promote blood vessel normalization to improve the hypoxic state within tumors, thus inducing enhanced RIT [54].

### 3.3. Organic Nanoparticles

Owing to its high biocompatibility and non-toxicity, liposomes have been used as nano-drug carriers in tumor treatment [55]. Liang et al. labeled a therapeutic radioisotope ^131^I onto albumin-encapsulated liposomes to improve vasculature permeability and promote the enrichment of drugs within tumor, so as to enhance the anti-tumor efficacy of radionuclides [56]. Chang et al. found that repeated administrations of the liposome-encapsulated ^188^Re nanoparticles increased its accumulation in the tumor tissues and bone marrow and prolonged the internal circulation time, thereby improving the killing effect of tumor cells [57]. In recent research, Kolašinac et al. intercalated radionuclide ^131^I into cationic fusogenic liposomes to form the novel nanocomposites. Compared with free iodine uptake, the usage of nanocomposites achieves a 10% increase in drug delivery efficiency with its special membrane fusion ability [55].

### 3.4. Chemotherapy and RIT

For instance, Tian et al. assembled paclitaxel onto human albumin pre-equipped with radionuclide ^131^I to form a novel nanocomposite ^131^I-HSA-PTX. In the animal tumor model, this nanocomposite showed strong tissue penetration ability, long internal circulation time, and the ability to inhibit the expression of HIF-1α so as to cause the improvement of hypoxia in tumor tissue and enhancement of tumor cell-killing effect [58]. In another instance, Sisin et al. compared the therapeutic effects of radionuclide ^192^Ir with or without bismuth oxide nanoparticles (BiONPs) and cisplatin in vitro. According to the combined treatment with radionuclide ^192^Ir, the experiment was divided into three groups: BiONPs, Cis, and BiONPs-Cis combination. Compared with the BiONPs and Cis treatment group, the breast cancer cell line (MCF-7) with BiONPs-Cis combination treatment showed the highest sensitization enhancement ratio (SER) of 4.29 [59]. Cytryniak et al. loaded doxorubicin and radionuclide ^177^Lu into the monoolein (GMO)-based lipid liquid-crystalline nanoparticles (cubosomes) to form a novel complex (DOTAGA-OA-^177^Lu). This complex had strong structural stability, and compared with pure nuclide therapy, it had stronger cell-killing effect in vitro [60]. 

### 3.5. PDT and RIT

For deep tumor therapy, radionuclides that can produce cerenkov luminescence (CL) can be used as the alternative excitation light sources to enhance the anti-tumor efficacy of photodynamic therapy (PDT) [61]. Wang et al. synthesized a novel nanocomposite ^131^I-ZGCs-ZnPcC_4_) including radionuclide ^131^I, tetrakis (4-carboxyphenoxy) zinc phthalocyanine, and Cr^3+^-doped zinc gallate. Radionuclide I-131 can activate ZGCs to produce stable and continuous luminescence so as to activate a photosensitizer (ZnPcC4) for photodynamic therapy. This synergistic anti- tumor effect had been confirmed both in vivo and in vitro [61]. In his recent work, Cai et al. labeled radionuclide I-131 into bovine serum albumin to form a complex (^131^I-BSA), which was then loaded into a prepared chlorin lipid nanovesicle to form a novel nanoplatform (^131^I-BSA@LCN-Apt). Via CL and ionizing radiation, radionuclide I-131 can activate photosensitizer Ce6 for PDT. The usage of this nanoplatform brought an excellent synergistic anti-tumor effect. The experimental results showed that a combined treatment of PDT and ^131^I-BSA@LCN-Apt can produce an excellent synergistic anti-tumor effect [62].

### 3.6. PTT and RIT

For instance, Song et al. labeled radionuclide I-131 into transferrin to form a complex, and then bound this complex to polypyrrole (PPy) nanoparticles. Radionuclide I-131 can be used for radionuclide therapy (RIT), and polypyrrole (PPy) can be used as an effective excitation material for photothermal therapy (PTT) due to its inherent high near-infrared (NIR) absorption capacity. Moreover, because of transferrin with tissue-specific binding capacity, the synthesized nanomaterials (PPy@Tf-^131^I) could effectively aggregate in tumor tissues; this effect had also been confirmed in vivo and in vitro [63]. In another instance, Xia et al. designed a new melanin nanoprobe (PMNs-II-813), which has the potential for PTT with its high NIR absorption capacity. This melanin nanoprobe could have two therapeutic functions: PTT and RIT, when labeled with ^131^I. In the mouse model of prostate cancer, combined treatment of PTT and RIT produced a significantly better tumor-suppressive effect than the single treatment model [64]. 

### 3.7. Targeted Therapy and RI

In a recent study, Fu et al. constructed a nanocomposite (FA-GEF-^90^Y-LPNP), including folic acid (FA), gefitinib (GEF), ^90^Y, and lipid–polymer with a core–shell structure. Gefitinib as an oral epidermal growth factor receptor tyrosine kinase inhibitor can inhibit the growth, metastasis, and angiogenesis of tumors, while promoting tumor cell apoptosis. Radionuclide ^90^Y is an ideal therapeutic radionuclide, with a half-life of 64 h. Compared with single treatment, FA-GEF-90Y-LPNP exhibited a better anti-tumor ability and tolerable toxicity in vivo and in vitro [65]. 

### 3.8. Trimodal Synergetic RIT

For instance, Wu et al. labeled Cetuxima (Cet), 5-Fu, and ^131^I into poly (ethylene glycol)-poly (lactic acid) (PEG-PLA) nanoparticles to form a nanocomposite (Cet-PEG-PLA-5Fu-^131^I). Cetuximab, as a monoclonal antibody against epidermal growth factor receptor, was an anti-tumor targeted drug. 5-Fu was used for chemotherapy, while ^131^I was used for RIT. In the xenograft mouse model, Cet-PEG-PLA-5Fu-^131^I nanoparticles exhibited longer internal circulation time and better tumor-specific binding ability, thereby producing better anti-tumor effects compared to Cet-PEG-PLA-5Fu or Cet-PEG-PLA-^131^I [66].

## 4. Nanomaterials for RT-Based Combination Therapy

RT-based combination therapy, such as concurrent chemoradiation and combined RT and PDT, modulated by nanoparticle platforms, has also shown significant synergistic effect in recent studies.

### 4.1. Nanomaterials for Combined Chemotherapy and RT

Concurrent chemo and radiation therapies (CRT) is a standard treatment for many solid tumors, especially locally advanced cancers [67]. Many chemotherapeutants, such as cisplatin, docetaxel, and 5-fluorouracil, have been identified as radiosensitizers to improve local control and survival rates, while potentially eradicating distant micrometastatic disease [68,69,70]. However, concurrent chemoradiation causes more serious side effects, compared with therapy alone or sequential RT and chemotherapy [71]. Utilizing the nanoparticles with certain functions, such as controlled drug-release function, presents huge opportunities to overcome these challenges and improve the effect of chemoradiotherapy of cancer [72].

#### 4.1.1. Inorganic Nanoparticles

It has been proved that different types of inorganic nanoparticles are able to improve the anti-tumor efficacy of chemoradiation, such as gold (Au) [73], platinum (Pt) [74,75], bismuth(Bi)-based, manganese dioxide (MnO_2_), and iodine(I)-based.

##### Noble Metal Nanoparticles

For instance, Mirrahimi et al. recently synergized some novel nanocomposite (ACA) including cisplatin (CDDP), gold nanoparticles (AuNPs), and alginate hydrogel. CDDP, as a common cytotoxic drug, was used for systemic chemotherapy. Gold nanomaterials can be used as radiosensitizers to enhance RT efficacy. Compared with chemoradiation, combined therapy of the newly synthesized nanocomposite and external radiation significantly inhibited the tumor growth in a mouse model of colon cancer [73]. In another work, Charest et al. encapsulated gold nanoparticles and carboplatin in liposome preparations to form a novel nanocomplex. The same dose of carboplatin and gold nanomaterials equipped on liposome nanomaterials can significantly inhibit tumor growth and enhance RT anti-tumor efficacy, while carboplatin or gold nanomaterials alone have no radiosensitization effect in vivo [74]. 

##### Mesoporous Nanomaterials

Mesoporous nanomaterials have been used as excellent drug carriers due to their low toxicity, high capacity, and easy degradation [23]. For instance, Chen et al. utilized polyethylene glycol (PEG) to modify mesoporous tantalum oxide (^m^Ta_2_O_5_) nanoparticles to form novel nanocomposites (^m^Ta_2_O_5_-PEG). These mTa_2_O_5_-PEG nanocomposite can effectively load and deliver chemotherapy drugs, such as doxorubicin (DOX). Ta as a high-Z element in ^m^Ta_2_O_5_-PEG/DOX nanoparticles can increase the X-rays deposition within tumor tissues and enhance the anti-tumor effect of radiation. The toxicity of DOX-loaded ^m^Ta_2_O_5_-PEG nanoparticles combined with RT was greatly reduced compared with free DOX of same dose combined with RT in vivo [76]. For another instance, Liu et al. recently loaded Bi-based mesoporous litchi-shaped Na_0.2_Bi_0.8_O_0.35_F_1.91_:20%Yb nanoparticles into amphiphilic PEG as a drug delivery carrier. This nanocomposite can stably load and slowly release chemotherapy drugs such as DOX. NBOF-PEG nanoparticles including high-Z element Bi can increase the X-ray absorption of tumor tissues and enhance the anti-tumor efficacy of radiotherapy [77].

##### Magnetic Nanoparticles

As a result of good biocompatibility and biodegradability, magnetic nanoparticles have been used as drug delivery carriers for tumor treatment [78,79,80]. In a recent study, Yang et al. developed a cisplatin-loaded, poly dopamine-coated and GE11 peptide-conjugated multi-functional theranostic system (GE11-PDA-Pt@USPIOs) based on poly acrylic acid-coated ultra-small superparamagnetic iron oxide nanoparticles (PAA@USPIOs). GE11-PDA-Pt@USPIOs can efficiently achieve the targeted delivery of loaded cisplatin to EGFR-positive tumor cells, thus improving tumor tissue hypoxia and enhancing the anti-tumor efficacy of chemoradiation [79]. In another recent study, Yang et al. developed a drug-delivery system with multifunctional graphene oxide (GO). This drug-delivery system can co-deliver chemotherapy drugs (5-Fu) and radiosensitizers, such as FePt magnetic nanoparticles. The cytotoxicity and radiosensitization of these nanocomposites were verified in vitro [80].

##### Metal–Organic Framework

**Figure 7:**
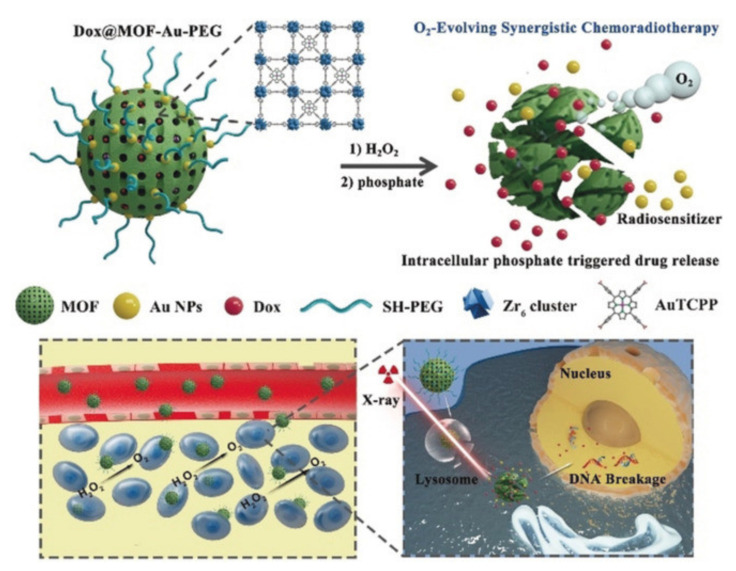
Preparation of DOX@MOF-Au-PEG and its mechanism for O_2_ self-supplying combined chemoradiotherapy. The AuNPs decorated on the surface of MOF effectively stabilize the nanocomposite and serve as radiosensitizers, whereas the MOF scaffold acts as a container to encapsulate chemotherapeutic drug doxorubicin. In vitro and in vivo studies verify that the catalase-like nanohybrid significantly enhances the radiotherapy effect, alleviating tumor hypoxia and achieving synergistic anticancer efficacy. Reproduced with the permission from He et al., Angew Chem. Int. Ed. Engl.; published by John Wiley and Sons, 2019.

Due to high storage capacities, compositions tailorability, biodegradability, and feasible modifiability, metal–organic framework (MOF) NPs have been tested as drug-delivery platforms for tumor treatment during some preclinical in vitro and in vivo experiments [81,82,83]. For instance, He et al. decorated porphyrinic MOF with gold nanoparticles to form a nanohybrid (Figure 7) [82]. Gold nanomaterials are used as RT sensitizers. MOF can deliver chemotherapeutic drugs. Porphyrins with peroxidase function can improve tissue hypoxia. These nanocomposites combined with RT significantly inhibited tumor growth with less toxicity in vivo and in vitro [82].

##### Rare Earth-Based Nanoparticles

It has been recently found that Ce-based nanoparticles have good radiosensitization effect via the photoelectric effect. Sun et al. constructed cisplatin-loaded LiLuF_4_:Ce^3+^ scintillation NPs (NP + Cis), which was synthesized by the crystal precipitation method and characterized by transmission electron microscopy (TEM). NP + Cis triggered massive DNA damage and effectively inhibited cell viability in vitro under X-ray radiation. The results of in vitro experiments showed that NP + Cis had higher biosafety, which could absorb enough irradiation and produce a synergistic inhibitory effect on tumor through the releasing Cis [84].

#### 4.1.2. Organic Nanoparticles

With the development of nanotechnology, numerous types of organic or polymeric nanostructures have been explored as drug delivery systems for concurrent chemoradiation. These nanoparticles usually have special hydrophobic/hydrophilic nanostructures, which can improve the bioavailability of chemotherapeutic drugs, especially insoluble drugs.

##### Nanoliposome

Due to its good tumor capillary permeability, nanoliposomes as drug delivery carriers can increase the accumulation of chemotherapeutic drugs in tumors and reduce their distribution in normal tissues, thus enhancing the efficacy and decreasing the toxicity of concurrent CRT [11,13,75,85,86]. Liu et al. conjugated hypoxic radiosensitizer nitroimidazoles with lipid molecules with a hydrolyzable ester bond to form MDH and then mixed MDH together with DSPE-PEG2000 and cholesterol to form a prodrug liposome (MLP). MLP was used to load and deliver doxorubicin (DOX) for chemotherapy. Since nitroimidazole improved hypoxia in tumor tissues, MLP can be used for radiosensitization and the delivery of chemotherapeutic drugs in a hypoxic microenvironment in vivo [86]. In another study, Zhang et al. loaded catalase (CAT) into liposomes constituted by a cisplatin (IV)-prodrug-conjugated phospholipid to form a CAT@Pt (IV)-liposome. CAT loaded into the liposome complex still retained enzyme activity, which can degrade hydrogen peroxide in the tumor to generate oxygen and improve tissue hypoxia, thereby enhancing the anti-tumor efficacy of chemoradiation. The combination of CAT@Pt (IV)-liposome and RT significantly inhibits tumor growth with low toxicity in vivo [13].

##### Organic and Polymeric Nanoparticles

Several polymeric nanomaterials, such as poly (ethylene glycol)-based, which are easily degradable and less toxic, have been widely developed as drug delivery carriers for chemoradiation [87,88,89,90]. 

For instance, Yin et al. introduced metronidazole (MN) moieties into the biodegradable polypeptide poly (ethylene glycol)-block-poly (l-glutamic acid) (PEG-b-PLG) to form novel nanocomposites (PEG-bP(LG-g-MN)), which can self-assemble into core–shell micelles in aqueous solution. PEG-b-P(LG-g-MN) micelles can effectively encapsulate DOX and release the drug quickly in a hypoxic microenvironment. Combined with low-dose X-ray therapy (4 Gy), PEG-bP (LG-g-MN) micelles effectively inhibited tumor tissue growth in vivo [87]. For instance, Zhang et al. modified PEG-PLGA nanoparticles with transferrin to make novel nanocomposites (Tf-NPs) for loading and delivering doxorubicin (DOX) and tetrahydrocurcumin (THC). Tf-NPs-DOX-THC with high drug loading efficiency and strong cytotoxicity for tumors can enhance radiotherapy sensitivity in vitro [89]. 

Some other organic polymeric nanomaterials have also been used to deliver drugs for chemoradiation [17,91,92]. In a recent study, Yao et al. loaded cisplatin prodrug (cisPt(IV))-conjugated phospholipid into perfluoro-15-crown-5-ether (PFCE) and used liposomes as stabilizers to prepare novel nanodroplets (PFCE@cisPt(IV)-Lip). PFCE@cisPt(IV)-Lip have good physiological stability and can perform tumor-targeting oxygen shuttling. Owing to the high oxygen loading capacity of PFCE, PFCE@cisPt(IV)-Lip can improve tumor tissue hypoxia and enhance radiotherapy sensitivity. Combined with RT, PFCE@cisPt(IV)-Lip could significantly inhibit the tumor growth and prolong the survival time of mice [92].

### 4.2. Nanomaterials for Combined PTT and RT

Photothermal therapy (PTT) utilizes heat generated by NIR optical absorbers to eliminate tumor cells [93,94]. The heat generated by PTT can promote tumor vasodilation and increase tumor blood supply, thereby improving hypoxia, which can get over the hypoxia-related RT resistance [35]. During the DNA synthesis phase, tumor cells are more sensitive to PTT, while the therapeutic advantage of RT lies in killing tumor cells in the mitotic phase, such as the G1 phase. Therefore, a combination of PTT and RT can effectively kill tumor cells in different phases [95]. In addition, PTT can lead to various DNA repair enzymes’ denaturation, thus significantly inhibiting the repair of DNA radiation damage and enhancing the anti-tumor effect of RT [95]. As a result, the RT therapeutic effect is effectively strengthened when combining PTT with RT.

Recently, noble metal nanoparticles, such as Au-based nanocomposites, have been extensively explored for the synergistic therapeutics of PTT and RT due to their strong NIR optical absorption [96,97,98,99,100,101,102]. For instance, Sun et al. wrapped gold nanorods with tumor cell membranes to form novel nanocomposites (GNR@Mem). GNR@Mem exhibited excellent light-to-heat transfer capability during the second NIR window period, which can increase the local temperature of tumor tissues and produce ROS, thereby effectively killing tumors. GNR@Mem encapsulated by the tumor cell membrane can remain stable in the microenvironment and possesses the capacity to target specific homologous tumor cells. Combined treatment of PTT and RT modulated by GNR@Mem significantly inhibited the proliferation of tumor tissues with no obvious side effect in vivo [101]. In another instance, Zhang et al. synthesized a novel nanocomposite BiPt-folic acid-modified amphiphilic polyethylene glycol (BiPt-PFA). The BiPt-PFA nanocomposite containing a Pt element has a strong NIR absorption capacity, which can produce photothermal effects. In addition, this synthetic nanocomposite can increase the X-ray absorption of tumor tissues, consume glutathione, and catalyze the decomposition of peroxides to generate oxygen to relieve hypoxia in TME. Therefore, BiPt-PFA may be used as an ideal material to mediate the microenvironment and promote the sensitivity of tumors to photothermotherapy and radiotherapy [102]. 

**Figure 8:**
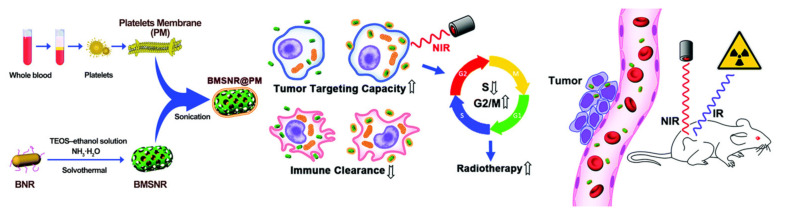
The preparation and mechanism of BMSNR@PM in vitro and in vivo. The PM camouflage reduced the endocytosis of BMSNRs by macrophages and enhanced their active tumor-targeting ability, resulting in a potent accumulation in the tumor site in vivo. BMSNR@PM altered the cell cycle of cancer cells in the presence of NIR; specifically, the proportions of the S and G2/M phases were lowered and increased, respectively, which contributed to a synergistic effect of NIR on BMSNR@PM-based radiotherapy. Reproduced with the permission from Chen et al., Biomater Sci.; published by Royal Society of Chemistry, 2019.

Due to their efficient heat generation abilities and X-ray attenuation coefficient, other heavy metal-based nanocomposites, such as Bi_2_Se and BiP_5_, have been developed as enhancers for synergistic thermo-radiotherapy [95,103,104,105,106]. For example, Zhou et al. developed an effective radiosensitizer, which was named bismuth heteropolytungstate (BiP_5_W_30_) nanoclusters. The BiP_5_W_30_ nanoclusters containing high-Z elements Bi and W can increase the consumption of glutathione, improve tumor tissue hypoxia, and increase X-ray deposition in tumor tissues, thereby enhancing the efficacy of radiotherapy. Reduced graphene oxide (rGO), as a NIR optical absorbance agent, can improve tumor blood perfusion in vivo and produce a photothermal effect. BiP_5_W_30_ nanoclusters coupled with rGO may form a novel and effective sensitizer for synergistic thermo-radiotherapy [106]. For another example, Chen et al. utilized a platelet membrane to camouflage mesoporous silica-coated bismuth nanorods to form a novel nanocomposite (BMSNR@PM). Platelet membrane camouflaging can endow the BMSNR@PM nanocomposite with immune escape ability and enhance the targeting capacity to tumor tissues. The BMSNR@PM nanocomposite had a strong near-infrared absorption capacity, which can produce a photothermal effect and enhance the blood supply of tumor tissues, thereby enhancing the RT efficiency. In a tumor-bearing mice model, the synergistic treatment of BMSNR@PMs and RT efficiently inhibited the growth of tumor tissue (Figure 8) [95]. Bao et al. loaded hafnium (Hf) clusters and manganese (III)–porphyrin ligands into the nanoscale metal–organic framework to form a new nanocomposite (fHMNM). The fHMNM containing catalase-like Mn(III)-porphyrin ligand can degrade hydrogen peroxide to oxygen, improve tumor tissue hypoxia, and enhance the sensitivity of PTT and RT. In mouse cancer models, synergistic thermo-radiotherapy modulated by fHMNM significantly inhibited the growth of tumor tissue, with no obvious toxic reaction [103].

Magnetic hyperthermia (MHT) refers to heat generated by alternating magnetic field (AMF) inductive mediators to eliminate tumor cells [107]. Recently, many magnetic nanoparticles act as the inductive mediators of alternating magnetic field (AMF) [78,108,109,110]. For instance, Wang et al. fabricated block copolymer micelles (polyethylene glycol-block-polycaprolactone) containing hyaluronic acid (HA) and Mn-Zn ferrite magnetic nanoparticles (MZF) via a two-step preparation. The receptor–ligand binding between HA and CD44 endows these synthetic nanocomposites with the capacity of active targeting to CD44-rich tumor cells. During exposure to AMF, MZF nanoparticles can generate hyperthermia, which can increase the blood supply of tumor tissues and overcome radioresistance caused by hypoxia. These synthetic nanocomposites have the ability to produce a magnetocaloric effect and radiosensitization at the same time, which was confirmed by experiments [110].

Many organic nanocomposites, such as polypyrrole (PPy), have been extensively applied to synergistic therapeutics of PTT and RT [111]. In a recent study, Zhou et al. loaded polypyrrole (PPy) onto γ-polyglutamic acid nanogels to synthesize a novel nanocomposite. These nanocomposites exhibited good water dispersibility and colloid stability. PPy with strong NIR absorption capacity can produce a photothermal effect and enhance RT outcomes. Cooperative PTT and RT modulated by these nanomaterials can enhance the therapeutic effect for solid tumors [111].

### 4.3. Nanomaterials for PDT with RT

Photodynamic therapy (PDT), with minimal invasiveness, low toxicity, and high selectivity, has been an alternative method for clinical cancer treatment in recent years [112]. In the presence of O_2_, photodynamic therapy utilizes photosensitizers activated by light of appropriate wavelength to generate ROS, which can further destroy tumor cells [112]. Recent studies have shown that PDT combined with RT increased the radiation sensitivity of tumors and improved the effectiveness of treatment while shortening the exposure time or reducing the radiation dose [113,114,115]. 

**Figure 9:**
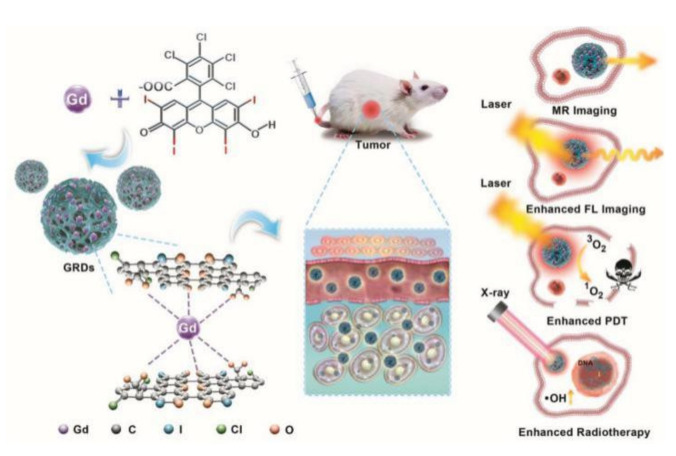
The preparation of GRDs and the mechanism of GRDs-based in vivo fluorescence/MR imaging-guided PDT and RT. GRDs showed unique absorption with a 7.7-fold fluorescence enhancement and 1.9-fold in singlet oxygen generation efficiency versus RB, which enables fluorescence imaging and PDT. Effective coordination between Gd ions and RB molecules led to GRDs with better T1-weighted MR imaging ability and enhanced radiotherapy. After the coordination, the GRDs showed good biocompatibility and could combine the PDT effect with radiosensitization under laser and X-ray irradiation, causing highly effective cancer cell death. Reproduced with the permission from Sun et al, Adv. Mater.; published by John Wiley and Sons, 2020.

The conventional combination therapy (PDT/RT) requires two kinds of excitation light sources, including the light at a specific wavelength and X-ray [116,117,118,119]. For instance, Sun et al. synthesized of Gd-rose bengal coordination polymer nanodots (GRDs). Compared with free rose bengal, GRDs exhibit higher luminous intensity and more effective singlet oxygen generation. The combination of PDT and RT modulated by the GRDs showed significant synergistic therapeutic effects that were verified in vivo and in vitro (Figure 9) [119]. PDT combined with radiotherapy has an intrinsic safety and a relatively positive radiosensitization effect. However, owing to reflection, scattering, and absorption, visible light has a poor ability to penetrate tissues, resulting in poor lethality of PDT on deep tumor tissues [114]. To solve this problem, Chen et al. designed novel nanoparticles with scintillation or glow persistently and loaded them with photosensitizers to form nanocomplexes for PDT in 2006. Under X-ray irradiation, these nanocomplexes delivered to the deep tumor tissue can scintillate and glow persistently, thereby activating the photosensitizer. Based on this research, a new photodynamic therapy strategy named X-ray-induced PDT was developed [120]. Zou et al. synthesized of Ce-doped lanthanum fluoride(III) (LaF_3_:Ce(III)) nanoparticles via a wet chemical method in dimethyl sulfoxide (DMSO), which was then encapsulated together with protoporphyrin IX (PPIX) as a photosensitizer into poly (D, L-lactide-co-glycolide) (PLGA) microspheres to form microspheres. Under X-ray irradiation, LaF_3_:Ce(3+)/DMSO nanoparticles can emit strong green fluorescence, which can activate the photosensitizer PPIX to induce oxidative stress and DNA damage [121]. Zhong et al. reportedly synthesized Ce-doped NaCeF_4_:Gd, Tb scintillating nanoparticles. Upon X-ray irradiation, these nanoparticles can emit fluorescence to activate a photosensitizer to kill tumor cells. Otherwise, due to the strong X-ray absorption of Ce and Tb ions, they can be also used as radiosensitizers. The excellent synergistic therapeutic effect of PDT and RT was confirmed in vitro and in vivo [122]. In another recent study, Noghreiyan et al. loaded TiO_2_ and PPIX in mesoporous silica nanoparticles. Upon X-ray irradiation, the wavelengths emitted by TiO_2_ nanoparticles overlap with the wavelengths of light to activate PPIX. After X-ray irradiation, these Ti-MSN/PPIX nanoparticles significantly inhibited cell proliferation in vitro [123].

Tumor hypoxia may significantly reduce the therapeutic efficacy of PDT and RT, since both of them rely on adequate oxygen supply [114,124]. In a recent study, Dan et al. synthesized ultra-small gold nanoclusters (Au NCs-ICG) loaded with green (ICG). These synthetic nanoclusters can decompose tumor endogenous hydrogen peroxide into oxygen to improve the tumor hypoxia, thereby enhancing the treatment sensitivity of PDT and RT. In addition, due to the inherent X-ray absorption ability of gold, Au NCs-ICG nanoclusters can enhance the anti-tumor effect of radiation [124].

In PDT, oxygen molecules are often converted into singlet oxygen (^1^O_2_) with high cytotoxicity due to the action of activated photosensitizer [125,126]. Singlet oxygen is rapidly metabolized in the body, and its diffusion range is limited, so that its damage range is limited to the subcellular structure where PS is located [127]. Therefore, the therapeutic effect of PDT is closely related to the subcellular structure of PS. Mitochondria, as the common location of photosensitizers in cells, have been considered as a potential therapeutic target of PDT. Ni et al. loaded cationic ruthenium (Ru) and photosensitizer Hf-porphyrin on nanoscale metal–organic frameworks (nMOFs) to form nanocomposites. Cationic ruthenium (Ru)-based PSs with strong targeting mitochondrial properties have recently been reported. In vivo, the synthetic nanocomplex exhibits characteristics of targeting mitochondria. Under irradiation, the photosensitizer would be activated to catalyze the production of ^1^O_2_, which damaged the mitochondrial membrane and induced cancer cell apoptosis [128].

### 4.4. Nanomaterials for Combined Genetic Therapy and RT

Genetic therapy refers to introducing genetic materials, such as microRNA (miRNA) or small interfering RNA (siRNA), into target tumor cells to regulate the expression of target genes or synthesize foreign proteins to kill tumor cells [129]. Recently, genetic therapy has become a powerful alternative strategy for tumor treatment. The number of studies about the use of nanoparticles as modulators to achieve the combination of RT and genetic therapy is growing rapidly.

SiRNA, which are silence-specific genes that encode tumor-related proteins, has been a promising therapeutic modality for solid tumors [130]. Kievit et al. designed a novel nanoparticle that can deliver therapeutic siRNAs into tumor cells. Therapeutic siRNA into tumor cells can downregulate the expression of DNA repair protein apurinic endonuclease 1 (Ape1), which enhanced the sensitivity of tumor cells to RT. In a mouse model of GBM, the degree of knockdown of Ape11 activity was significantly negatively correlated with the survival time of mice [131]. In another recent study, Yong et al. synthesized gadolinium-containing polyoxometalate coupled chitosan nanospheres (GdW_10_@CS). These nanospheres can stimulate and mediate HIF-1a siRNA to knockdown the expression of HIF-1α and inhibit the self-healing of DNA. In addition, GdW_10_@CS nanospheres promoted the consumption of glutathione and improved tumor tissue hypoxia, thereby enhancing the anti-tumor efficacy of radiotherapy [132]. Erel-Akbaba et al. combined cyclic peptide iRGD into solid lipid nanoparticles. These synthetic nanoparticles can deliver siRNAs against EGFR and PD-L1 into tumor cells of glioblastomas (GM), causing the downregulation of EGFR and PD-L1 expression so as to achieve targeted therapy and immunotherapy. The combination of these nanoparticles and RT significantly inhibited the GM tumor growth and prolonged the survival time of mice [133].

The targeted delivery of specific genes with anti-tumor effects via nanoparticles has been an alternative therapeutic modality to increase radiotherapy sensitivity and kill tumor cells [134,135]. For instance, Gaca et al. combined membrane heat shock protein specific antibody (cmHsp70.1) with human albumin nanoparticles. These conjugates can be combined with miRNAs that target the apoptotic protein survivin inhibitor to form a novel nanocomposite (Hsp70-miRNA-NP). This delivery mediated by nanomaterial can increase the uptake of miRNAs in tumor cells, thus reducing the survival rate of cloned cells and enhance its radiation sensitivity in vitro [134]. In another instance, Chen et al. screened a novel DNA repair inhibitor named leucine-rich repeat-containing protein 31 (LRRC31). This protein was identified through genome-wide CRISPR screening. The delivery of LRRC31 genes into tumor cells through nanomaterials can upregulate LRRC31 gene expression. The upregulation of LRRC31 expression can inhibit the repair of DNA double-strand break so as to increase the sensitivity to RT in mice bearing breast cancer metastasis [135].

### 4.5. Nanomaterials for Combined Immunology Therapy and RT

In recent years, immunotherapy (IT) has become a powerful new generation oncology therapy strategy. Immunotherapy drugs are designed to activate patients’ own immune system to attack tumor cells [136,137]. IT alone has demonstrated the potential to affect durable and adaptable control for some cancers with specific pathological types. However, most patients did not benefit significantly from IT alone due to low immune activation effect and treatment-related toxicities [138]. An increasing body of evidence suggests that IT combined with other standard therapies, such as radiotherapy, has provided increased immune recognition and durable response, which can lead to improved survival [139]. 

Nanomaterials acting as modulators to achieve the combination of IT and RT offer opportunities to reduce immune-related toxicity, augment immune response, and enhance anti-tumor efficacy [140,141,142,143,144]. In a recent study, Pang et al. reported that polysaccharide nanoparticles (ANPs) induced immune response by dendritic cells activation, which could amplify the abscopal effect of radiotherapy. ANPs significantly inhibited the growth of primary and metastatic tumor tissue in tumor-bearing mice receiving RT [140]. In another recent study, Yu et al. synthesized bismuth sulfide nanoparticles (BiNP) conjugated with ganoderma lucidum polysaccharide (GLP). The compound (GLP-BiNP) can enhance immune response by activated dendritic cells (DC) and enhanced DC maturation. In addition, GLP-BiNP can be used as a radiosensitizer due to high X-ray absorption of the Bi element [141]. Min et al. formulated novel nanoparticles (AC-NPs), which can capture tumor-associated antigens and deliver them to antigen-presenting cells (APCs). In the B16F10 melanoma model, AC-NPs amplified RT induced an ‘abscopal effect’ and significantly enhanced the anti-tumor efficacy of αPD-1 [142]. 

Immunoadjuvants (IA) have become promising tools for cancer IT. Via activating APCs, it can induce innate or adaptive immune responses [145]. IA include in situ vaccines, oncolytic viruses, chemokines antagonists, and device activated agents [133]. The combination of RT and IA can not only improve the immune response of tumor tissues but also enhance the RT-mediated abscopal effect [146,147]. Luo et al. developed a safe and effective nanovaccine combined with RT, which activated the stimulator of interferon genes pathway to augment T cell responses. The combination of RT and this nanovaccine significantly increased the percentage of CD8^+^ T cells in tumor tissues and inhibited tumor growth [148].

### 4.6. Trimodal Synergetic RT

Recently, RT-based trimodal synergistic therapies such as chemotherapy/PTT/RT, chemotherapy/immunotherapy/RT, and PTT/PDT/RT have been gradually used for tumor treatment [149,150,151,152].

#### 4.6.1. Chemotherapy/PTT/RT

Recently, Sun et al. constructed a doxorubicin-loaded gold nanocage coated with a tumor cell–erythrocyte hybrid membrane (CM-EM-GNCs@DOX) for NIR-activated chemotherapy/PTT/RT. It concurrently kept the excellent homologous target ability and immune evasion capability, which contribute to the nanoparticles’ more efficient accumulation and lower clearance speed within tumors. Under a specific wavelength range of NIR irradiation, CM-EM-GNCs@DOX generated a strong photothermal effect, which not only destroyed the nanostructure to release DOX for more controllable precise chemotherapy but also improved RT efficiency [153]. More recently, Alamzadeh et al. constructed a multifunctional nanoplatform, which is made up of alginate hydrogel encapsulating gold nanoparticles and cisplatinum (ACA). The experimental data showed that ACA-based tri-modal therapy could lead to a much better anticancer efficacy when compared with single or bi-modality strategies. The level of ROS within KB cells receiving tri-modal therapy was 4.4 times higher than that in control group cells. It also proved the downregulation of Bcl-2 anti-apoptotic factor and the upregulation of a Bax pro-apoptotic factor through analyzing related gene expression [154].

#### 4.6.2. Chemotherapy/Immunotherapy/RT

Gao et al. developed one type of nanoparticles containing Se, which could deliver the DOX to tumor sites through the EPR effect after intravenous injection. In the meantime, radiation stimuli can induce the quick release of DOX and enhancement of chemotherapy. To be specific, radiation could oxidize diselenide-containing NPs to seleninic acid, which can activate NK cells to achieve the synergistic antitumor effect and immunomodulatory function. Maybe the above Se-based NPs could act as a potential approach for the combination of chemotherapy, immunotherapy, and RT [48]. Au et al. constructed antibody-mimic selective high-affinity ligand-functionalized nanoparticles loading DOX to treat tumors with overexpressed human leukocyte antigen-D related (HLA-DR) antigen. In addition to releasing chemotherapeutics DOX to directly kill tumor cells, NPs can upregulate the surviving cells’ expression of HLA-DR, which can further induce enhanced immunogenic cell death. Otherwise, the released Dox also sensitizes the cancer cells to irradiation by inducing cell cycle arrest and preventing the repair of DNA damage. In vivo biodistribution and toxicity studies confirm that the targeted NPs enhanced tumor uptake and reduced systemic toxicities of DOX [155]. 

#### 4.6.3. PTT/PDT/RT

Xu et al. synthesized the Au nanocages modified by hyaluronic (AuNCs-HA), which possessed the following several functionalities: First of all, AuNCs-HA can be used as a good contrast agent to enhance RT because of its excellent LSPR peak in the NIR region. Secondly, such a nanoplatform can efficiently absorb high-energy radiation such as X-ray and generate Auger electrons, thus having potential as a radiosensitizer. At last, due to its high specific surface area and good photocatalytic property, AuNCs-HA can act as the photosensitive agent for PDT. Compared with one treatment method alone, PTT/PDT/RT had the significantly enhanced ability of eliminating tumors and inhibiting tumor growth [156]. Liu et al. recently developed a mesoporous heterostructure UCNP@NBOF-FePc-PFA, which is made up of Lu-doped upconversion nanoparticles and Bi-based nanomaterial loading iron phthalocyanine. It can achieve PTT/PDT/RT triggered by X-ray and NIR. The nanohybrid can be further modified. The finally obtained nanoplatform has strong X-ray absorption and photothermal effect, good upconversion luminescence capacity, and available NIR and X-ray dual-triggered ROS generation ability. The above features can be used in clinical upconversion luminescence or CT bioimaging [157]. 

#### 4.6.4. Targeted Therapy/Chemotherapy/RT

Fu et al. developed an injectable thermosensitive nanosystem loading Se-based nanoparticles and sorafenib (SOR) for the localized synergistic chemoradiotherapy to patients with hepatocellular carcinoma. The experimental data showed that the continuous release of SOR from this nanosystem was observed with hydrogel degradation for a prolonged time. The combination of localized chemotherapy and RT could reduce the expression of Ki67 and CD34 and activate the caspase-3 signal pathway in HepG2 cells to promote the apoptosis [65]. Bikhezar et al. also tried to utilize polymeric nanocarriers encapsulating MEK162 (binimetinib, a MEK1/2 inhibitor) for overcoming the blood–brain barrier (BBB). The nanosystem successfully crossed the BBB in the in vitro model and thus has the ability to deliver therapeutics drugs to brain tumor sites. When combined with temozolomide (TMZ) and RT to treat glioma spheroids, it could efficiently inhibit tumor growth [158].

#### 4.6.5. Other Synergistic Treatment

Song et al. constructed tumor hypoxia-targeting multifunctional nanoparticles (CPTA), which can make use of the special microenvironment of hypoxia for cancer treatment. Due to the composition of CPTA, it can achieve not only the simultaneous PTT and PDT generated by Ce6 but also tirapazamine’s (TPZ) sensitization of chemotherapy and RT. During PDT, the oxygen was consumed, thus leading to exacerbated hypoxia in tumor sites, which can subsequently greatly enhance chemotherapy sensitized by TPZ and cause a synergistic anti-tumor effect. It was also proved by both in vitro and in vivo experiments [159]. 

## 5. The Clinical Translation of Nanomaterials as Radiosensitizers

In the past twenty years, as a result of emerging advanced nanomaterials and nanobiotechnology’s quick development, various promising approaches for cancer diagnosis and treatment have arisen continuously [160]. Many types of nanomaterials and related nanomedicine have been applicated clinically to improve the treatment outcome of cancer patients [161,162]. In recent years, many clinical studies for nanoparticles as radiosensitizers have been carried out one after another (Table 2 and Table 3).

The French company Nanobiotix developed a first-in-class 50 nm radio-enhancer named NBTXR3, whose effective ingredient is crystalline hafnium oxide (HaO_2_) with surface functionalized with the negatively charged phosphate [162,163]. HaO_2_ is the oxide of hafnium (Z = 72) and has a density as high as 9, both of which make it an efficient radiosensitizer [164]. NBTXR3 is physically and chemically inert in biological media, which provided a safety perspective for its biomedical use. When NBTXR3 enters the tumor tissues and receives RT, its high electron density makes the interaction with incoming radiation more likely to occur, thus causing a higher energy deposited within irradiated tissues than RT alone and more subsequent cancer cells death. Preclinical studies have proven that the utilization of NBTXR3 during RT has a strong anti-tumor efficacy and might be likely to improve patient outcomes in many types of cancer [164,165]. The phase 1 study of NBTXR3 showed that the combination of NBTXR3 with RT is a feasible therapeutic method, which can yield ideal radiological and pathologic responses in patients with locally advanced soft tissue sarcoma [164]. What is more, in the multicenter, randomized controlled phase II/III study (NCT02379845), receiving the treatment of NBTXR3 and RT have showed clinically meaningful advantage compared with being treated with RT alone [166]. Hoffmann et al. recently found that intratumoral injection of NBTXR3 followed by IMRT was viable and demonstrates a good biosafety in elderly or weak patients with locally advanced head and neck squamous cell carcinoma (HNSCC) [167]. Moreover, there are still a few ongoing clinical studies of NBTXR3 [168].

In 2013, Tillement et al. constructed a new Ga-based nanoparticle agent, with the name of AGuIX and the diameter of sub-5 nm; it is also able to be quickly cleared through the kidneys [169]. AGuIX’s ability to enhance RT is mainly achieved through the component of Gd (Z = 64), which has the potential to cause a strong interaction with X-rays and improve RT efficacy [169,170,171]. Due to the EPR effect, AGuIX’s nanosize is helpful to its efficient accumulation in tumor tissue, finally causing significantly improved RT response [172]. Recent studies in vitro and in vivo have also demonstrated AGuIX’s outstanding radiosensitizing properties [170]. Nowadays, two clinical trials for AGuIX NPs combined with RT are ongoing. Preliminary experimental results of phase I clinical trials (NCT02820454) showed treating patients suffering brain metastases with the combination of AGuIX and RT is safe [173,174]. The more precise assessment of its efficacy will be accomplished in the ongoing phase II studies. Otherwise, in phase 1 clinical trials (NCT03308604) that combine it with chemoradiation or brachytherapy to treat patients with locally advanced cervical cancer, AGuIX’s safety and tolerance doses are being evaluated [175].

It has been found that some traditional chemotherapy drugs can serve as radiosensitizers to increase the efficiency of RT through different mechanisms [68,69,70]. For example, paclitaxel (PTX) can arrest cells at the G2/M phase, which is more sensitive to radiation [68]. However, severe hypersensitivity reactions caused by solvents and peripheral neurotoxicity might influence the treatment intensity when using the generic PTX combined with RT [176]. The reaction of PTX and serum albumin in high-pressure homogenization enables successfully synthesizing nanoparticle albumin-bound paclitaxel (nab-PTX), so as to avoid the use of toxic solvent [177]. Compared with free paclitaxel, nab-PTX possesses significantly improved solubility, less infusion time, and a lower risk of peripheral neuropathy or hypersensitivity reactions [178]. Recently, Kaira et al. verified that weekly cisplatin and nab-PTX treatment combined with concurrent RT is feasible for patients with NSCLC [179]. The results of another clinical trial also showed that biweekly nab-PTX and carboplatin with the combination of RT shows good systemic anti-tumor activity for the patients mentioned above, without observing significant intolerance. Further studies are needed to assess the efficacy of this strategy [180].

As we all know, camptothecin can act as a potent inhibitor for topoisomerase I and HIF-1α. In the meantime, as an investigational nanoparticle drug, camptothecin-based CRLX101 has a diameter in the range of 20 to 30 nm, and its zeta potential is slightly negative [181]. In some preclinical studies, CRLX101 was regarded as a radiosensitizer in murine xenograft models and colorectal cancer cell lines because it can inhibit the activation of HIF-1α caused by radiation [182]. It is also found that there seems to be no intolerance in patients suffering locally advanced rectal cancer when treated with CRLX101’s combination with standard capecitabine-based CRT [183].

## 6. Conclusions

Nanomaterials have been widely used in enhancing RT due to their unique physical and chemical properties. In general, nanomaterials can improve tumor’s sensitivity to RT mainly in the following ways. (1) Under irradiation, nanomaterials containing high atomic number elements can generate a large number of free radicals and other active species in tumor sites through the photoelectric effect, Auger effect, ionization effect, and other effects, to damage the DNA of tumor cells. (2) Nanoparticles with tumor-targeting ability can improve RIT or realize synergetic anti-tumor therapy combining RT with other therapeutic strategies by delivering therapeutic radioisotopes or other therapeutic agents to the tumor (such as photothermal conversion reagents, chemotherapeutics, photosensitizers). (3) Nanomaterials can also load H_2_O_2_ enzymes to induce the decomposition of endogenous H_2_O_2_ in tumors, thus improving the oxygenation of tumor parts and thereby overcoming the hypoxia-related RT tolerance. (4) Nanomaterials can enhance RT by regulating the cell cycle and making tumor cells in the G2/M phase, which is more sensitive to RT. (5) Nanomaterials can induce the accumulation of radiation-related DNA damage and eventually lead to cell apoptosis by inhibiting the DNA self-repair mechanism in tumor cells as well.

Although recent advances in nanotechnology have improved the efficiency of RT to some extent, there are still a number of related problems to be solved to achieve the desired effect. First of all, the selection of radiation intensity and dose in RT must be considered carefully, as different kinds of rays mean different energy and different killing ability to tumor cells. An excessive dose or intensity of radiation may cause pain and side effects that the patients cannot withstand. On the contrary, too small a dose or intensity of radiation cannot effectively kill tumor cells, and it could even enhance the radiation resistance of the tumor. Therefore, the selection of an appropriate radiation intensity and dose is very important. Secondly, a considerable number of present RT sensitizers are based on noble metals such as gold and platinum, which greatly limits their wide use in clinical cancer treatment due to the rare reserves and high prices. At last, although nanomaterials used for sensitizing RT often have high biocompatibility and low toxicity, the long-term toxicity caused by its accumulation in the human body must be carefully considered due to the individuals’ different tolerance and removal capacities of nanomaterials.

## Figures and Tables

**Table 1:** Some examples of nanomedicine for enhanced RT. Caelyx, liposomal doxorubicin; NLE-CDDP, nanoliposome encapsulated cisplatin; MLP, hypoxic radiosensitizer-prodrug liposome; MPEG-PCL/PTX polymeric micelles, methoxy poly(ethylene glycol)-poly(ε-caprolactone)/paclitaxel polymeric micelles; DLN, drug-loaded nanocarrier; HA-Fe-NIs-DOX, hyaluronic acid-amphiphilic ferrocenium-hexane-nitroimidazole-doxorubicin; Cetuximab-IONPs, magnetic iron-oxide nanoparticles bioconjugated to cetuximab; MnO_2_-functioned ANPs-PTX, manganese dioxide-functioned albumin bound paclitaxel nanoparticles; FA-GSJNs-DOX, folic acid-Au-mesoporous silica Janus-doxorubicin-NPs; SeC@MSNs-Tf/TAT, selenoamino acid@multifunctional mesoporous silica nanoparticles-transferrin/TAT cell-penetrating peptide; mTa_2_O_5_-PEG/DOX, mesoporous tantalum oxide-polyethylene golycol/doxorubicin. Reproduced with the permission from Yang et al, Nano Res; published by John Wiley and Sons, 2020.

Nanomaterials	Nanocarriers	Drug	Cancer Type	Ref.
Liposomes	Caelyx	Doxorubicin	Human osteosarcoma	[10]
	NLE-CDDP	Cisplatin	Lewis lung carcinoma	[11]
	MLP	Doxorubicin	Glioma	[12]
	CAT@Pt(IV)- liposome	Catalase and cisplatin(IV)	Breast cancer	[13]
Micelles	DLN	Paclitaxel	Glioblastoma multiforme	[14]
	MPEG-PCL/ PTX polymeric micelles	Paclitaxel	Human cervical carcinoma	[15]
	HA-Fe-Nis-DOX	Doxorubicin	Prostate cancer	[16]
Other organic nanoparticles	DOC-NPs	Docetaxel	Gastric cancer	[17]
	Albumin-bound paclitaxel	Paclitaxel	Ovarian adenocarcinoma or mammary carcinoma	[18]
Inorganic nanoparticles	CetuximabIONPs	Cetuximab	Glioblastoma	[19]
	MnO_2_-functioned ANPs-PTX	Paclitaxel	Colon cancer	[20]
	FA-GSJNs-DOX	Doxorubicin	Hepatocellular carcinoma	[21]
	SeC@MSNsTf/TAT	Selenoamino acid	Cervical cancer	[22]
	mTa_2_O_5_- PEG/DOX	Doxorubicin	Breast cancer	[23]

**Table 2:** Ongoing clinical trials of combination of nanomedicine and RT for malignant tumors.

NCT Number	Study Title
NCT01946867	NBTXR3 and Radiation Therapy in Treating Patients with Locally Advanced SCC of the Oral Cavity or Oropharynx
NCT04505267	NBTXR3 and Radiation Therapy for the Treatment of Inoperable Recurrent Non-Small Cell Lung Cancer
NCT03589339	NBTXR3 Activated by Radiotherapy for Patients with Advanced Cancers Treated with An Anti-PD-1 Therapy
NCT04484909	NBTXR3 Activated by Radiation Therapy for the Treatment of Locally Advanced or Borderline-Resectable Pancreatic Cancer
NCT04615013	NBTXR3, Chemotherapy, and Radiation Therapy for the Treatment of Esophageal Cancer
NCT04862455	NBTXR3, Radiation Therapy, and Pembrolizumab for the Treatment of Recurrent or Metastatic Head and Neck Squamous Cell Cancer
NCT04834349	Re-irradiation With NBTXR3 in Combination with Pembrolizumab for the Treatment of Inoperable Locoregional Recurrent Head and Neck Squamous Cell Cancer
NCT04789486	Nano-SMART: Nanoparticles with MR Guided SBRT in NSCLC and Pancreatic Cancer
NCT03308604	AGuIX Gadolinium-based Nanoparticles in Combination with Chemoradiation and Brachytherapy
NCT03818386	Radiotherapy of Multiple Brain Metastases Using AGuIX^®^
NCT02901483	A Study of PEP503 With Radiotherapy in Combination with Concurrent Chemotherapy for Patients with Head and Neck Cancer
NCT02465593	A Study of PEP503(Radio-enhancer) with Radiotherapy and Chemotherapy for Patients with Rectal Cancer

**Table 3:** Completed clinical trials of combination of nanomedicine and RT for malignant tumors.

NCT Number	Study Title
NCT01433068	NBTXR3 Crystalline Nanoparticles and Radiation Therapy in Treating Patients with Soft Tissue Sarcoma of the Extremity
NCT02379845	NBTXR3 Crystalline Nanoparticles and Radiation Therapy in Treating Randomized Patients in Two Arms with Soft Tissue Sarcoma of the Extremity and Trunk Wall
NCT02820454	Radiosensitization of Multiple Brain Metastases Using AGuIX Gadolinium Based Nanoparticles
NCT01652079	CRLX101 in Combination with Bevacizumab for Recurrent Ovarian/Tubal/Peritoneal Cancer
NCT01380769	A Phase 2 Study of CRLX101 (NLG207) in Patients with Advanced Non-Small Cell Lung Cancer
NCT00333502	Study of CRLX101 (NLG207) in the Treatment of Advanced Solid Tumors
NCT01612546	Pilot Trial of CRLX101 in Treatment of Patients with Advanced or Metastatic Stomach, Gastroesophageal, or Esophageal Cancer That Cannot be Removed by Surgery
NCT02187302	CRLX101 (NLG207) in Combination with Bevacizumab for Metastatic Renal Cell Carcinoma (mRCC) Versus Standard of Care (SOC)
NCT01625936	CRLX101 Plus Bevacizumab in Advanced RCC
